# Impact of Rocket Launch and Space Debris Air Pollutant Emissions on Stratospheric Ozone and Global Climate

**DOI:** 10.1029/2021EF002612

**Published:** 2022-06-24

**Authors:** Robert G. Ryan, Eloise A. Marais, Chloe J. Balhatchet, Sebastian D. Eastham

**Affiliations:** ^1^ Department of Geography University College London London UK; ^2^ Yusuf Hamied Department of Chemistry University of Cambridge Cambridge UK; ^3^ Laboratory for Aviation and the Environment Department of Aeronautics and Astronautics Massachusetts Institute of Technology Cambridge MA USA

**Keywords:** ozone depletion, black carbon, radiative forcing, space tourism, rockets, GEOS‐Chem

## Abstract

Detailed examination of the impact of modern space launches on the Earth's atmosphere is crucial, given booming investment in the space industry and an anticipated space tourism era. We develop air pollutant emissions inventories for rocket launches and re‐entry of reusable components and debris in 2019 and for a speculative space tourism scenario based on the recent billionaire space race. This we include in the global GEOS‐Chem model coupled to a radiative transfer model to determine the influence on stratospheric ozone (O_3_) and climate. Due to recent surge in re‐entering debris and reusable components, nitrogen oxides from re‐entry heating and chlorine from solid fuels contribute equally to all stratospheric O_3_ depletion by contemporary rockets. Decline in global stratospheric O_3_ is small (0.01%), but reaches 0.15% in the upper stratosphere (∼5 hPa, 40 km) in spring at 60–90°N after a decade of sustained 5.6% a^−1^ growth in 2019 launches and re‐entries. This increases to 0.24% with a decade of emissions from space tourism rockets, undermining O_3_ recovery achieved with the Montreal Protocol. Rocket emissions of black carbon (BC) produce substantial global mean radiative forcing of 8 mW m^−2^ after just 3 years of routine space tourism launches. This is a much greater contribution to global radiative forcing (6%) than emissions (0.02%) of all other BC sources, as radiative forcing per unit mass emitted is ∼500 times more than surface and aviation sources. The O_3_ damage and climate effect we estimate should motivate regulation of an industry poised for rapid growth.

## Key Points

- Air pollutant emission inventory for current space sector and future tourism input to a coupled chemistry and radiative transfer model
- Upper stratospheric Arctic ozone loss from launch chlorine and re‐entry nitrogen oxide emissions undermines Montreal Protocol success
- Warming efficiency of space tourism (soot) emissions about 500‐times greater than surface and aircraft sources of soot

## 1 Introduction

The space industry is one of the world's fastest growing sectors. Global revenue generated from this industry is forecast to grow from 350 million USD in 2019 to more than 1 trillion USD by 2040 (Morgan Stanley, 2020). This demand stems from significantly reduced launch costs driven by commercialization (Jones, 2018), increased reliance on satellite technologies for global positioning systems, surveillance and broadband internet (Alvino et al., 2019; Dolgopolov et al., 2018; George, 2019), and postulated space resource extraction (Hein et al., 2020) and militarization (Quintana, 2017). To meet growing demand, new spaceports and launch vehicle companies are being established in historically aeronautically active nations such as the US and Russia, and in nations with emerging space sectors such as China and India (Patel, 2019; Roberts, 2019). In 2021, commercial space flights by Virgin Galactic (Gorman, 2021), Blue Origin (Johnson, 2021), and SpaceX (Wattles, 2021) demonstrated that space tourism is plausible, though the scale of this nascent industry is uncertain. Such rapid growth demands detailed understanding of the potential impact on the protective stratospheric ozone (O_3_) layer and climate.

Orbital rockets require multiple stages to achieve thrust through the Earth's atmosphere. At the end of each stage, spent booster or rocket stages separate from the central launch vehicle and are either discarded or reused. Propulsion is achieved with a fuel and an oxidizer (collectively the “propellant”). The four most common fuels are kerosene, hypergolic fuels, liquid hydrogen (cryogenic), and solid fuels. Combustion emissions common to all propellants include water vapor (H_2_O) and nitrogen oxides (NO_x_ ≡ NO + NO_2_) (Dallas et al., 2020). Other pollutants include black carbon (BC) from carbon‐based solid and hypergolic fuels and kerosene, and alumina particles (Al_2_O_3_) and gaseous chlorine from solid fuels (Dallas et al., 2020). Rockets are unique among anthropogenic sources, due to direct injection of pollutants to all atmospheric layers. Crewed and reusable rockets, historical space debris and discarded rocket components also emit thermal NO_x_ on re‐entry through the mesosphere (Larson et al., 2017; Park & Rakich, 1980).

Almost all these emitted pollutants deplete stratospheric O_3_ via gas‐phase reactions or by promoting heterogeneous chlorine (Cl)‐activated O_3_ loss on aerosol or cloud surfaces (Ross et al., 2009). Cl depletes O_3_ and Al_2_O_3_ enhances Cl‐activated O_3_ loss by an order of magnitude more than an equal mass of stratospheric sulfate aerosols (Danilin, Ko, et al., 2001, Danilin, Shia, et al., 2001; Jackman et al., 1998). Direct injection of H_2_O to the stratosphere may enhance O_3_ loss via gas‐phase reactions or by contributing to formation of polar stratospheric clouds (PSCs) (Kirk‐Davidoff et al., 1999). Concerted measurement and modeling studies in the 1980s and 1990s determined that complete O_3_ destruction occurs in the wake of plumes of solid and kerosene propelled rockets (Ross et al., 2000), but that this local effect is negligible on a global scale compared to O_3_ destruction by the dispersed emissions (Danilin, Shia, et al., 2001; Prather et al., 1990). Global depletion of stratospheric O_3_ determined to first order and with early generation chemistry transport models (CTMs) is small (0.01%–0.1%) in comparison to O_3_ depleting substances like chlorofluorocarbons (1%–2%) (Braesicke et al., 2018; Danilin, Ko, et al., 2001; Jackman et al., 1996, 1998; Prather et al., 1990; Ross et al., 2009). Based on these studies, launch rates would need to increase by at least a factor of 10 to match the impact of regulated O_3_ depleting substances (Braesicke et al., 2018; Ross et al., 2009). The space sector has evolved substantially since these estimates were obtained to include private companies and national and regional space agencies in Asia, The Middle East, Europe, and Australasia with an increasing proportion of launches in the tropics and subtropics (Kyle, 2020).

Assessment of O_3_ loss due to thermal NO_x_ emissions from heating of space debris and reusable components as these re‐enter the atmosphere is limited. There has been substantial build‐up of space debris and increased use of reusable rockets, though Larson et al. (2017) determined that annual launches of reusable rockets would need to reach 100,000 for re‐entry heating NO_x_ from reusable stages to cause a 0.5% decline in global stratospheric O_3_. This is three to four orders of magnitude more than annual re‐entries by the SpaceX Falcon 9 reusable boosters. Even so, space debris is a pressing concern due to recent exponential growth in the amount of debris in orbit (ESA, 2021). Lengthening orbital lifetimes due to anthropogenically driven cooling and consequent contraction of the upper atmosphere may increase space debris collisions (Brown et al., 2021) leading to more uncontrolled re‐entry heating emissions. The uncertain size of the space tourism industry is also a concern, as the passenger vehicles and orbital launch reusable rocket stages produce NO_x_ on re‐entry.

Short‐lived climate forcers emitted by rockets also offset the radiative balance of the atmosphere, predominantly due to absorption of incident shortwave solar radiation by BC from kerosene and other hydrocarbon‐based fuels. This instantaneous radiative forcing was determined by Ross and Sheaffer (2014) to first‐order to be 16 ± 8 mW m^−2^ in the stratosphere due to a year of emissions from a fleet of rockets burning equal amounts of kerosene, hypergolic, and cryogenic fuels. Dominated by BC (70%) from kerosene combustion. The remainder (28%) was due to solid rocket emissions of Al_2_O_3_ absorbing more upwelling longwave radiation than the incoming sunlight reflected by the particles. Warming by the greenhouse gases H_2_O and CO_2_ was minor (Ross & Sheaffer, 2014).

The space sector remains unregulated by international treaties such as the Montreal Protocol (Ross et al., 2009) and the global impact of air pollutant emissions from rocket exhausts and re‐entry heating of heat shields, spent rockets, and space junk on atmospheric composition and climate is yet to be assessed with a detailed, global, 3D CTM for the modern space sector and for plausible space tourism offerings. Here we compile an inventory of air pollutant emissions from recent (2019) rocket launches, and re‐entries of reusable and discarded rocket components and reported space debris, as well as for a speculative space tourism industry. We implement these in the global GEOS‐Chem CTM coupled to the Rapid Radiative Transfer Model for Global climate (RRTMG) to determine the effect of rocket launch and re‐entry heating emissions on stratospheric O_3_ and global radiative forcing.

## 2 Methods

### 2.1 Emission Inventory Development

The number of rocket launches per year has increased steadily since a lull in the mid‐2000s, from 58 launches in 2003 to over 100 launches in 2018 and 2019 (Figure S1 in Supporting Information S1), an average increase of three launches each year or 5.6% a^−1^. To create an emissions inventory of modern‐era rocket launches, we compiled details of the timing, geolocation, and rocket mass of all 2019 launches and crewed re‐entries from the Space Launch Report database (Kyle, 2020) and the timing, geolocation, and mass of reported re‐entering debris and spent upper rocket stages from The Aerospace Corporation (2020). Additional details are in the Supporting Information S1.

We use a standard approach to calculate emissions of pollutants from rocket launches in 2019, that is, the product of the activity factors (amount of fuel burned at each rocket stage) and reported emission factors of pollutants for the types of fuel used. The latter, also termed emission indices, are reported as the mass of pollutant emitted per mass of fuel burned. We reasonably assume that all fuel is utilized at each launch stage. Even the Falcon 9 reusable first stage rocket only reserves ∼6% of total fuel mass for controlled re‐entry and landing (Kim et al., 2021). Re‐entry heating NO_x_ emissions from returning crewed spacecraft and rocket components should be parameterized using re‐entry velocity, trajectory, surface area and mass (Park et al., 2021), but only mass is readily available. We determine NO_x_ emissions equivalent to 17.5% of the mass of each returning reusable component. This is consistent with Larson et al. (2017) and is based on estimates for NASA Space Shuttle re‐entries (Park & Rakich, 1980). We model complete vapourization of rocket stages discarded above 50 km during launch, controlled payload re‐entries and unplanned re‐entry of space debris, resulting in NO_x_ emissions equivalent to 100% of the mass of the re‐entering object. Additional organic and inorganic pollutants form due to the complex range of chemical matrices of rocket propellants and parts and extreme temperatures during both launch and re‐entry (Park et al., 2021), but there are only reported emission factors for the most common air pollutants (chlorine, BC, H_2_O, Al_2_O_3_ and NO_x_).

We also calculate emissions for a speculative scenario of annual space tourism offerings by Virgin Galactic, Blue Origin and SpaceX. Only Virgin Galactic has announced plans to offer 400 flights each year (Sheetz, 2020). Given this, we determine emissions for daily suborbital launches by Virgin Galactic and Blue Origin and weekly orbital launches by SpaceX. Virgin Galactic includes an aircraft that reaches 14 km altitude using standard jet fuel, before the rocket (spaceplane) burns a hybrid propellant of solid rubber (hydroxyl‐terminated polybutadiene or HTPB) fuel and liquid nitrous oxide oxidizer producing NO_x_, H_2_O and BC. The single stage Blue Origin rocket burns liquid hydrogen and oxygen and so emits H_2_O and NO_x_. SpaceX uses a two‐stage Falcon 9 series rocket (Table S1 in Supporting Information S1) that burns kerosene fuel emitting NO_x_, H_2_O and BC. We also include re‐entry heating NO_x_ emissions as 17.5 mass % of the Blue Origin pod and SpaceX orbital capsule and first stage reusable rocket and complete burn‐up (100 mass %) of the Falcon 9 Stage 2 rocket. All space tourism launches are modeled to occur in the morning local solar time at the demonstration launch sites in New Mexico for Virgin Galactic, Texas for Blue Origin, and Florida for SpaceX.

### 2.2 Implementation of Launch and Re‐Entry Emissions in GEOS‐Chem

We use GEOS‐Chem version 12.9.3 (https://doi.org/10.5281/zenodo.3959279, accessed 8 August 2020) coupled to RRTMG to simulate global atmospheric composition due to rocket launch and re‐entry heating emissions. The model is run at 4° latitude × 5° longitude (∼400 × 500 km) horizontal resolution over 47 vertical layers from the Earth's surface to the lower mesosphere (0.01 hPa; ∼80 km). The model is driven with the NASA offline Modern‐Era Retrospective analysis for Research and Applications version 2 (MERRA‐2) meteorology and includes coupled HO_x_‐NO_x_‐VOC‐O_3_‐halogen‐aerosol tropospheric chemistry. Stratospheric chemistry is represented with the Unified tropospheric‐stratospheric Chemistry eXtension (UCX) scheme (Eastham et al., 2014). We add Al_2_O_3_ as a transported tracer and include in UCX the heterogeneous Cl‐activation reaction on Al_2_O_3_ between chlorine nitrate (ClONO_2_) and hydrochloric acid (HCl), forming nitric acid (HNO_3_) and diatomic chlorine (Cl_2_) with a reaction probability (γ) of 0.02 (Molina et al., 1997). Mesospheric chemistry in GEOS‐Chem includes simple linearized chemistry for O_3_ (McLinden et al., 2000), and monthly mean production and loss rate constants for other trace gases (Murray et al., 2012). Gravitational settling of BC follows Stoke's law (Eastham et al., 2014). We do not account for complete O_3_ loss in the wake of rocket plumes (Ross et al., 2000), as the effect on global O_3_ is at least an order of magnitude less than O_3_ depletion due to global long‐term accumulation of rocket pollutants (Danilin, Ko, et al., 2001). The RRTMG code calculates the top‐of‐the‐atmosphere shortwave and longwave direct instantaneous radiative forcing due to attenuation of radiation by trace gases and aerosols throughout the atmospheric column (Heald et al., 2014). BC is represented in GEOS‐Chem‐RRTMG with a dry geometric radius of 0.020 μm and refractive index of 1.95–0.79i at 550 nm. Mie scattering code is then used to calculate relative humidity and wavelength dependent aerosol optical properties. Additional details of RRTMG are provided by Heald et al. (2014).

To implement rocket launch emissions in GEOS‐Chem, we assume that the launch trajectory is at the same longitude and latitude as the launch coordinates and that emissions are instantaneous. Based on inspection of Space Shuttle launch trajectories (NASA, 2011), launches only typically deviate significantly from the launch coordinates at 70–80 km altitude, close to the model ceiling. The launch is also brief (2–3 min) compared to the model emission timestep (60 min). For the 2019 and SpaceX space tourism orbital launches, only the boosters and first stage rocket emissions are assumed to occur within the altitude range of the model. Booster emissions extend to ∼50 km and the first stage rocket to the top of the model (80 km). Suborbital space tourism emissions from Blue Origin and Virgin Galactic spacecraft stop at ∼50 km, the altitude at which the rockets cease burning fuel (Kordina, 2021). We use the launch profiles of mass of propellant burned in Ross and Sheaffer (2014) to represent the vertical distribution of rocket launch air pollutant emissions in GEOS‐Chem.

Heterogeneous reaction of Cl on Al_2_O_3_ is only appreciable for particles with diameters in the medium (0.01–1 μm) size range, as these can remain suspended in the atmosphere for several years (Danilin, Ko, et al., 2001; Ross et al., 2009). To account for this, we only represent heterogeneous Cl activation for 8% of total Al_2_O_3_ mass emitted, based on the mass contribution of the medium size range of these particles from the Athena II solid fueled rocket (Schmid et al., 2003). We assume an effective radius of 0.14 μm for this chemically relevant Al_2_O_3_. For simplicity, we distribute all launch emissions from the 3 failed Iranian rocket launches into the lowest model layer and assume the same emission factors as for a successful launch, as these rockets are reported to have exploded at the launch site (Brumfiel, 2019; Kyle, 2020).

Re‐entry NO_x_ emissions from space debris and rocket components discarded during launch are distributed evenly across the top two model layers (60–80 km altitude). We assume that the emissions from re‐entry are localized to the grid box corresponding to the reported latitude and longitude. The latitudes and longitudes for re‐entry of space debris are often coarsely reported to within 200 km, but this is well within the model resolution (400–500 km). We assume for simplicity, and given the relatively coarse model resolution, that spent rocket components re‐enter and burn up in the same horizonal grid square as the launch site.

The standard version of the model uses prescribed methane (CH_4_) concentrations that would lead to spurious results when assessing the influence of rocket emissions on CH_4_. We follow the approach of Kerkweg et al. (2006) of sampling monthly mean CH_4_ pseudo fluxes from a GEOS‐Chem simulation that uses prescribed monthly mean global surface concentrations obtained by interpolating NOAA flask measurements for 1983–2016 and extrapolated to 2019 (Murray, 2016). We then use these pseudo fluxes to determine the response of CH_4_ to perturbations in oxidants such as Cl and hydroxyl (OH) radicals that influence its abundance, and to capture long‐term feedbacks on O_3_ concentrations.

We spin up the model without space sector emissions for 7 years (2012–2018) prior to 2019 to accommodate slow transport and turnover rates in the stratosphere (Engel et al., 2017). The three simulations we sample are (a) a decade without rocket emissions (baseline), (b) a decade of contemporary rocket emissions, beginning with 2019 emissions followed by nine years of growth in these at 5.6% a^−1^, and (c) the first 3 years of simulation (b) with our speculative scenario of space tourism emissions that are the same in each year. Three years of space tourism offerings are simulated to accommodate the time it takes for O_3_ chemistry in the stratosphere to equilibrate (NASA, 2011; Ross et al., 2009). All simulations use the same 2019 meteorology to isolate the effect of emissions on atmospheric composition. We use the baseline simulation to assess the effect of sustained growth in rocket launches and a speculative space tourism industry on stratospheric O_3_ and radiative forcing. In what follows, we refer to the 2019 rockets and the current 5.6% a^−1^ launch growth rate as contemporary and our speculative scenario as space tourism. Even though the atmosphere is agnostic to the intent of each rocket launch, this distinction is to aid in directing international regulation.

## 3 Results and Discussion

### 3.1 Inventory of Air Pollutant Emissions From Rocket Launches and Re‐Entry Heating

**Figure 1:**
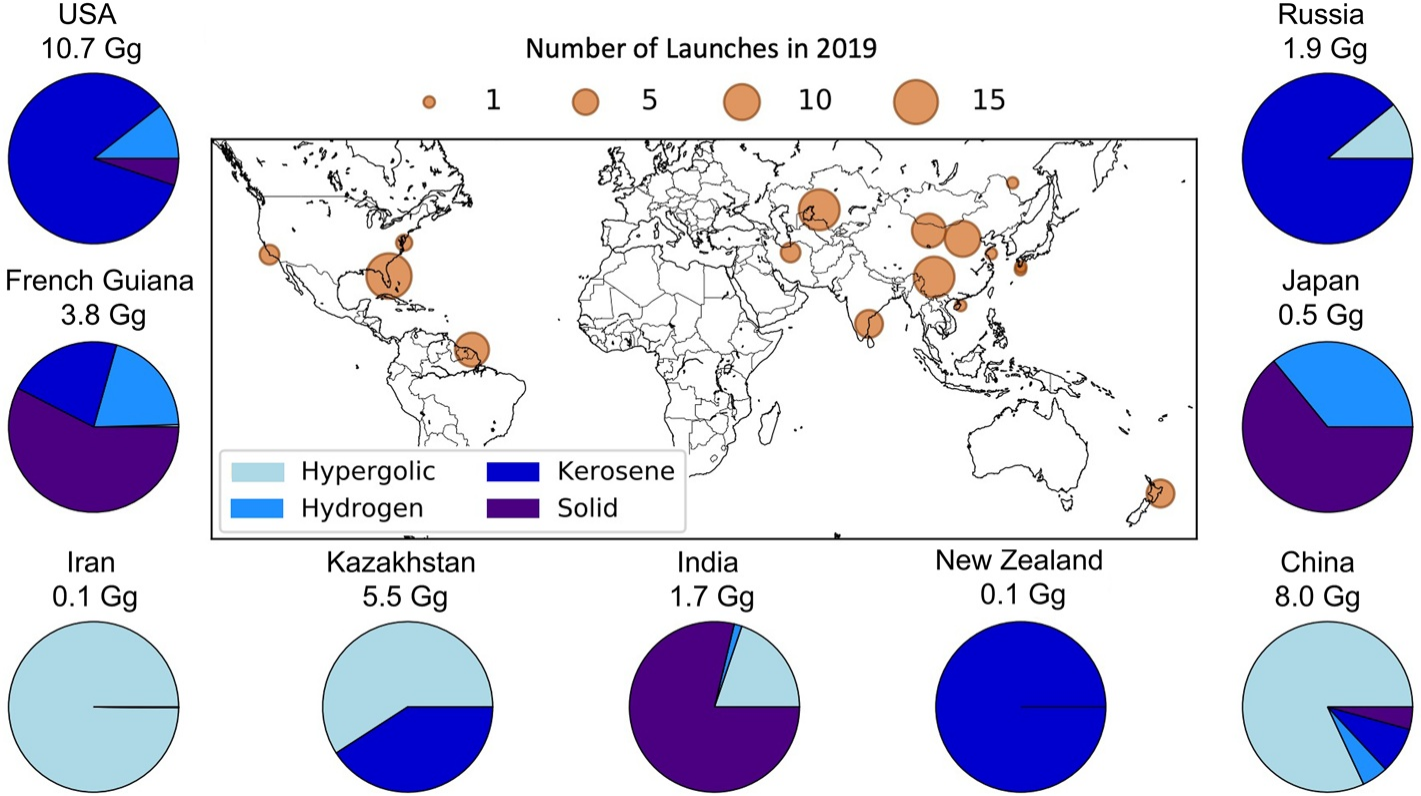
Locations and fuel types of rocket launches in 2019. Marker size in the map indicates the number of launches at each location. Pie charts indicate the proportion of the four main fuel types at each launch location. Numbers above each pie chart are total propellant mass used in each country. Additional details are in Table S1 in Supporting Information S1.

The map in Figure 1 shows the locations of all 103 documented 2019 rocket launches, dominated by China (34 launches), Russian‐operated launches in Russia and Kazakhstan (22), and US‐operated launches in the US (21) and New Zealand (6). Others include European Space Agency (ESA) launches from French Guiana in South America (9), and launches in India (6) and Japan (2) by their respective space agencies. A detailed summary of the characteristics of all 2019 launches is in Table S1 in Supporting Information S1. The pie charts in Figure 1 show the proportion, by mass, of the four main fuel types (kerosene, hypergolic, liquid hydrogen and solid) used in each country. Solid fuels dominate launches from Japan, India and French Guiana. Hypergolic fuels are typical in China, Kazakhstan and Iran. Kerosene is the dominant fuel for launches in New Zealand, Russia and the US. The 32 Gg total rocket propellant used in 2019 includes 45% kerosene, 32% hypergolic, 14% solid and 8% liquid hydrogen.

The emission factors we use to calculate air pollutant emissions are summarized in Table 1 for NO_x_ (as NO), H_2_O, BC, Al_2_O_3_ and gaseous chlorine. The latter is emitted mostly as HCl, but includes some Cl that accounts for rapid conversion of HCl to Cl in the wake of the rocket (Prather et al., 1990). Oxidation of NO in high temperature rocket plumes forms products such as HNO_3_, with HNO_3_:NO ratios between 0.6 and 1.3 for solid rocket plumes (Popp et al., 2002). These ratios are not known for all fuel types, so we represent all NO_x_ as NO. The aircraft used by Virgin Galactic burns Jet A fuel. To estimate emissions for this, we use reported emissions factors of 13 g NO kg^−1^, 0.04 g BC kg^−1^ and 4.35 kg H_2_O kg^−1^ (Brink, 2020; Phillips, 2020). We assume emission factors for HTPB are the same as those for the other hydrocarbon‐based fuel, kerosene. This may be conservative, as Ross and Sheaffer (2014) suggest HTPB emission factors are double those for kerosene.

**Table 1:** Emission Factors of Dominant Pollutants From Rocket Fuel Types ^a^

Fuel	Emission factors [g kg^−1^]					
	NO_x_	H_2_O	BC	HCl	Cl	Al_2_O_3_
Kerosene/HTPB	14^b^	300^c^	35^c^ ^,^ ^d^			
Hypergolic^e^	20^d^	550^c^ ^,^ ^d^ ^,^ ^f^	4^c^			
Liquid hydrogen	33^b^	1000^c^ ^,^ ^d^ ^,^ ^f^				
Solid	3^f^	370^b^ ^,^ ^c^ ^,^ ^d^	4^c^	210^f^	3^f^	380^f^

^a^ Means are given where more than one estimate is reported in the literature.

^b^ Larson et al. (2017).

^c^ Ross and Sheaffer (2014).

^d^ Ross et al. (2009).

^e^ Hypergolic propellants typically use hydrazine‐based fuel.

^f^ Federal Aviation Administration (2005).

**Figure 2:**
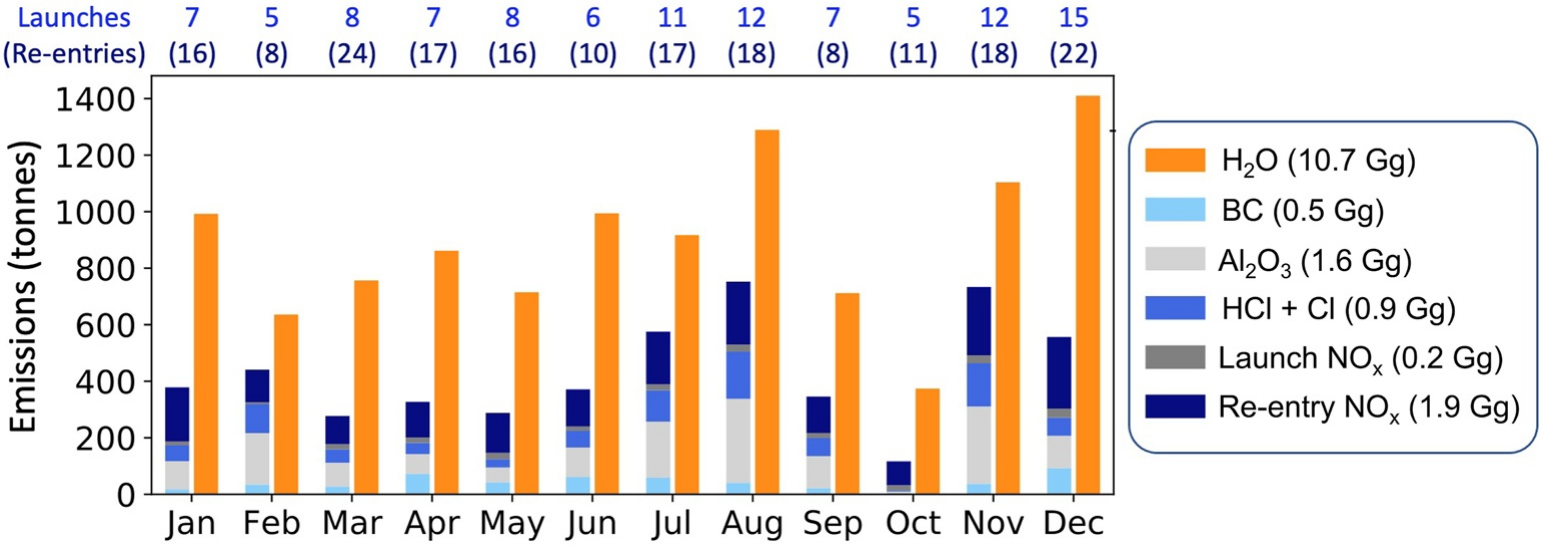
Monthly space sector emissions in 2019 from boosters, first launch stages and re‐entry burn as implemented in GEOS‐Chem. Numbers in the legend give the total annual emissions of each pollutant. Listed above the chart are the number of launches and re‐entries in each month. Emissions increase to 16.7 Gg H_2_O, 0.8 Gg BC, 2.5 Gg Al_2_O_3_, 1.4 Gg HCl + Cl and 3.4 Gg NO_x_ in the last year of the 10‐year simulation.

Figure 2 shows the mass of each pollutant emitted in each month in 2019 within the altitude range of the model. This includes combustion of fuel by the booster and first stage rocket and from re‐entry heating. The relative mass of each emitted component is similar in each month. The booster and first stage rocket emissions that occur within the altitude limits of GEOS‐Chem account for the majority of total emissions from all stages: 80% for NO_x_, 94% for Al_2_O_3_ and HCl + Cl, 84% for H_2_O, and 79% for BC. The amount released above 15 km is 78%–79% for H_2_O and BC, and 68%–69% for Cl and Al_2_O_3_ (Figure S2 in Supporting Information S1). Most NO_x_ is from re‐entry burn, so the majority (95%) is emitted above 60 km (Figure S2 in Supporting Information S1). The absolute and proportional contribution of re‐entry heating NO_x_ emissions in our inventory is likely conservative, as the geolocated re‐entries included in the inventory are only half of all re‐entries cataloged for 2019 in ESA's publicly accessible database (Figure S3 in Supporting Information S1 (ESA, 2021)). Annual space tourism emissions from the 782 launches and associated re‐entries total 30.4 Gg H_2_O, 2.1 Gg NO_x_ (33% re‐entry, 67% launch), and 1.0 Gg BC; much greater than the 2019 emissions for H_2_O (by a factor of 3) and BC (double). Combined annual re‐entry NO_x_ emissions for 2019 and our space tourism scenario total 4.2 Gg, similar to the lower‐end of annual 2–40 Gg NO_x_ emissions from burn‐up of meteorites that we infer from the equivalent mass range of cosmic dust (Plane, 2012).

### 3.2 Impact of Contemporary Rocket Launches and Re‐Entries on Stratospheric Chemistry and Radiative Forcing

**Figure 3:**
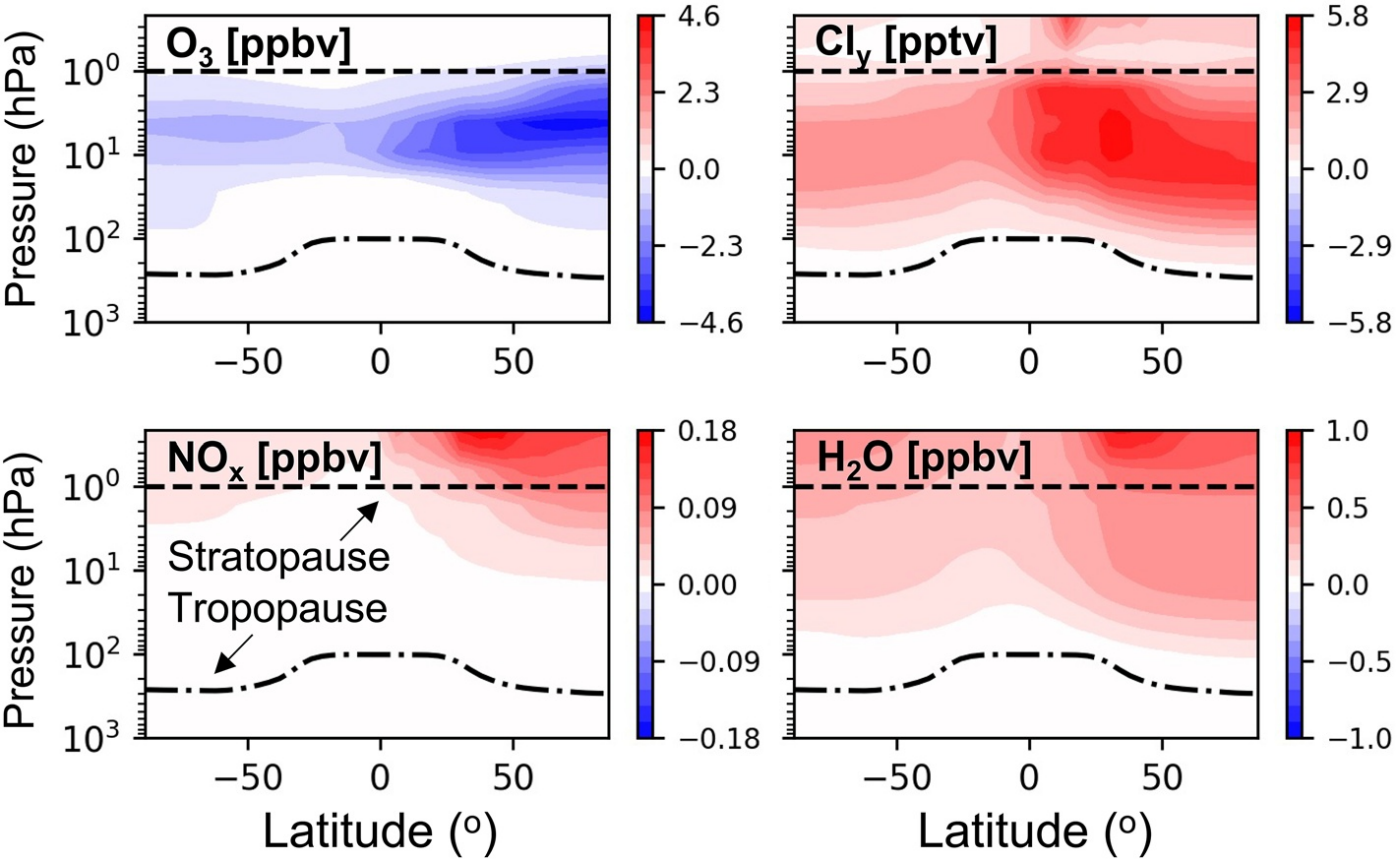
The effect of a decade of sustained growth in rocket and re‐entry burn emissions on atmospheric composition. Panels show the change in annual zonal mean mixing ratios of O_3_, Cl_y_, NO_x_, and H_2_O due to launch and re‐entry heating emissions.

Figure 3 shows zonal changes in annual mean O_3_, NO_x_, H_2_O, and total inorganic chlorine (Cl_y_ ≡ Cl + 2Cl_2_ + 2Cl_2_O_2_ + ClNO_3_ + ClO + ClOO + OClO + BrCl + ICl + HOCl + HCl) following a decade of contemporary rocket launch and re‐entry heating emissions. Global stratospheric O_3_ declines by 0.010% (or 0.034 DU). In a solid rocket plume measured by Ross et al. (1997) 30% of chlorine was Cl_2_. We found in a sensitivity simulation that stratospheric O_3_ abundance was relatively insensitive to emitting 35% chlorine mass as Cl_2_ instead of HCl. The different in O_3_ between the 2 simulations is <0.5%, due to rapid cycling between reactive chlorine species.

Our global modeled stratospheric O_3_ depletion is ∼200‐times less than the 2.2% depletion in 2018, relative to pre‐1980 levels, attributable to surface sources of ozone depleting substances (Braesicke et al., 2018). This is also less than half the amount ascribed to annual contemporary‐at‐the‐time solid and liquid rocket fuel emissions estimated by Ross et al. (2009) using a simple linear model, but similar to the 0.014% decline in global stratospheric O_3_ determined by Jackman et al. (1996) for 12 solid fuel rockets using a 2D CTM. Our simulation has 31 solid fuel rockets. The estimate by Jackman et al. (1996) excluded Cl activation by Al_2_O_3_, that, in a later study, increases their estimate of stratospheric O_3_ depletion to 0.025% (Jackman et al., 1998). This value was adjusted down to 0.015% when Danilin, Shia, et al. (2001) accounted for the effect of size distribution on the atmospheric lifetime of Al_2_O_3_. Peak decline in GEOS‐Chem O_3_ of 4.5 ppb (0.09%) occurs in the northern hemisphere upper stratosphere (∼5 hPa; ∼40 km), above the altitude range where O_3_ depletion is dominated by heterogeneous chemistry on PSCs (15–25 km; Solomon (1999)). A decade of rocket H_2_O emissions only causes <0.001% increase in PSC optical depth over the Arctic and Antarctic (Figure S4 in Supporting Information S1). The Antarctic spring was anomalously warm in 2019 (Wargan et al., 2020). We find that Antarctic spring O_3_ loss following 3 years of launch and re‐entry emissions is enhanced by just 7% in the portion of the stratosphere where PSCs are prevalent (100–10 hPa; Figure S4 in Supporting Information S1) for a simulation with 2019 emissions and 2020 meteorology relative to a simulation with both 2019 emissions and meteorology. The maximum Cl_y_ increase is 0.22% and occurs in the mid‐stratosphere (Figure 3). Peak increase in NO_x_ of 6.0% occurs in the lower mesosphere due to re‐entry heating emissions. H_2_O increase is also largest in the mid‐high latitude upper stratosphere and lower mesosphere, up to 0.010%, due to a long photochemical lifetime (Abbas et al., 1996) enabling long‐range transport upwards via Brewer‐Dobson circulation.

**Figure 4:**
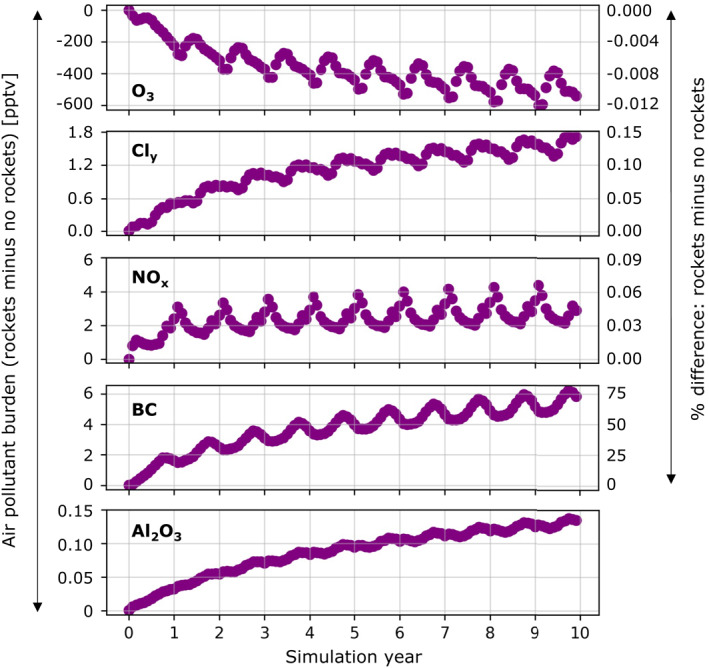
Influence of a decade of contemporary rocket launch and re‐entry heating emissions on stratospheric chemical composition. Points are GEOS‐Chem global monthly mean differences in abundance of O_3_, Cl_y_, NO_x_, BC and chemically active Al_2_O_3_ (see text for details) averaged over 200 to 1 hPa. Axes are absolute (left; all) and relative (right; all except Al_2_O_3_ as Al_2_O_3_ in the no‐rockets simulation is zero) changes. Seasonal cycles for O_3_ at the poles (60–90°) are in Figure S5 in Supporting Information S1.

Figure 4 shows the temporal evolution of changes in global mean stratospheric (200‐1 hPa) composition due to a decade of 5.6% a^−1^ growth in contemporary launch and re‐entry emissions. The strong seasonal cycles in O_3_ depletion and increases in Cl_y_, NO_x_, BC and Al_2_O_3_ take 2–3 years to equilibrate, consistent with earlier estimates of the stratospheric lifetime of rocket emissions (NASA, 2014; Ross et al., 2009). Peak decline in stratospheric O_3_ is in the spring, coincident with maximum enhancements in Cl_y_ and 1–2 months after the wintertime NO_x_ peak. In the polar (60–90°) upper stratosphere maximum O_3_ loss reaches 0.15% in the north and 0.04% in the south (Figure S5 in Supporting Information S1). The NO_x_ seasonal peak is in winter in both polar hemispheres (0.60% in the north; 0.15% in the south) due to the longer lifetime of NO_x_ in dark, cold conditions. The seasonal cycle of stratospheric H_2_O change follows that of NO_x_. More rocket launches and a greater number (and mass) of geolocated re‐entering objects are in the northern hemisphere, causing the January global peak in NO_x_ in Figure 4. The particles (Al_2_O_3_, BC) exhibit the same month‐to‐month variability. The major loss pathway for these particles is transport to the midlatitude and polar troposphere followed by deposition (Kravitz et al., 2012). As deposition at the poles peaks in summer and most rocket launches are in the northern hemisphere, BC and Al_2_O_3_ in Figure 3 peak in autumn‐winter. Weaker variability and smaller stratospheric burden for Al_2_O_3_ is because of more efficient deposition of much larger Al_2_O_3_ particles (0.14 μm compared to 0.02 μm for BC).

**Figure 5:**
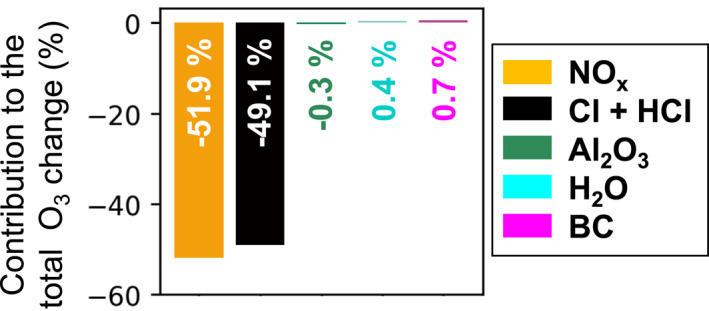
Contribution of individual pollutants to stratospheric O_3_ depletion. Bars and inset values show percent contribution of individual pollutants to the total change averaged from 200 to 1 hPa, determined as the percent difference in GEOS‐Chem simulations with all emissions and with emissions of a single air pollutant after 3 years of 2019 rocket launch and re‐entry heating emissions.

We also conduct sensitivity simulations to determine the relative contribution of individual emitted air pollutants to depletion of stratospheric O_3_. The results are in Figure 5 for year 3 of the simulations to accommodate the time it takes for stratospheric chemistry to equilibrate (Figure 4). NO_x_, mostly from re‐entry heating, accounts for the majority of O_3_ decline (51%), followed closely by chlorine from solid fuel rockets (49%). This result is in contrast to the Ross et al. (2004) finding that chlorine is the dominant contributor to O_3_ depletion, as their estimate did not account for re‐entry NO_x_. Our re‐entry heating NO_x_ emissions cause a decline in stratospheric O_3_ of 0.005%. This is 42‐times more than 0.00012% due to all exhaust emissions from 10 hypergolic fueled rockets (Ross et al., 2004) and 0.0001% inferred by Carpenter et al. (2018) for re‐entry objects in 2017. We only include 185 geolocated objects in our simulation (Section 3.1). If we were able to include NO_x_ emissions from all 351 objects re‐entering in 2019 (ESA (2021); Figure S3 in Supporting Information S1), stratospheric O_3_ loss due to re‐entry heating, to first order, would be ∼0.01%. The number of returning objects in 2019 is still far fewer than the 100,000 re‐entries that Larson et al. (2017) calculated to cause 0.5% O_3_ loss.

In our simulation, the effect of Al_2_O_3_ on stratospheric O_3_ (Figure 5) is 200‐times less than the effect of chlorine; a much greater difference than the factor of 4–6 difference obtained by Danilin, Shia, et al. (2001) using a 2D chemistry mechanism within a 3D atmospheric transport model. Warming of the stratosphere by BC leading to enhanced reaction kinetics and loss of O_3_ is not captured in GEOS‐Chem. Ross et al. (2010) found that this effect was potentially very significant, but that the response of the atmosphere to warming by BC is complex and highly variable.

**Figure 6:**
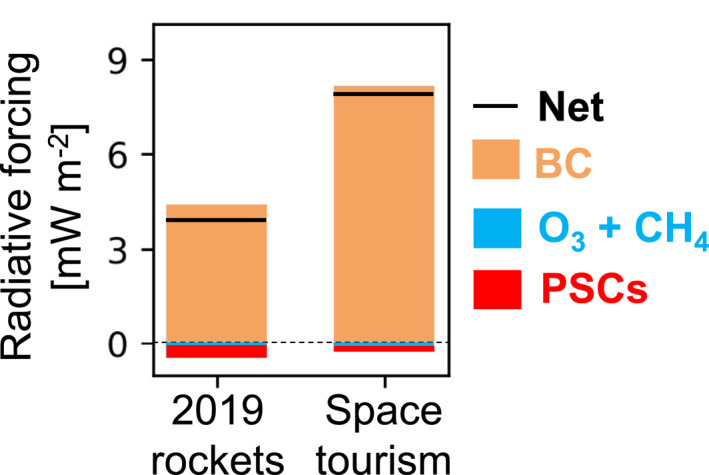
Effect of rocket launch and re‐entry emissions on global climate forcing. Bars show the GEOS‐Chem‐RRTMG top‐of‐the‐atmosphere instantaneous radiative forcing of BC (orange), combined O_3_ and CH_4_ (blue) and polar stratospheric clouds (PSCs, red) after a decade of growth in 2019 emissions (left) and after 3 years of constant space tourism emissions and growth in 2019 emissions (right). The black solid line is the net effect of all forcers.

Figure 6 shows the top‐of‐the‐atmosphere radiative forcing due to a decade of sustained growth in contemporary rocket launch and re‐entry emissions. Global mean forcing is +3.9 mW m^−2^, mostly due to BC (+4.4 mW m^−2^). The BC radiative forcing is dominated by the shortwave component (+4.3 mW m^−2^). Depletion of O_3_ and CH_4_ cause a small negative forcing (−0.016 mW m^−2^). A small (0.002%) global decline in CH_4_ in year 10 is due to its increased oxidation by Cl. The remainder of the negative forcing (−0.20 mW m^−2^) is due to H_2_O emissions enhancing formation of reflective PSCs (Figure S4 in Supporting Information S1). The largest forcing occurs over the northern high latitudes (regional mean, 60–90° latitude, +7.7 mW m^−2^) and the Antarctic (+5.1 mW m^−2^) (Figure S6 in Supporting Information S1). The global mean forcing due to BC we obtain (+4.4 mW m^−2^) is less than the +11 mW m^−2^ due to BC determined to first order by Ross and Sheaffer (2014), partly because their BC emissions (1.5 Gg) are three times more than our 2019 BC emissions (0.5 Gg; Figure 2). They also estimated a forcing of +4.5 mW m^−2^ due to Al_2_O_3_ particles, due to greater absorption of longwave radiation than reflection of shortwave radiation. We do not model Al_2_O_3_ radiative forcing, as the phase, optical properties and size distribution of Al_2_O_3_ from solid rockets are poorly constrained (Ross & Sheaffer, 2014). The BC radiative forcing we calculate is 3.2% of the global total due to BC of +139 mW m^−2^ (Dong et al., 2019); much greater than the proportional contribution (∼0.01%) of contemporary rocket emissions to global total BC emissions (6.7 Tg a^−1^; Dong et al. (2019)).

### 3.3 Impact of Proposed Space Tourism on Stratospheric Ozone and Radiative Forcing

**Figure 7:**
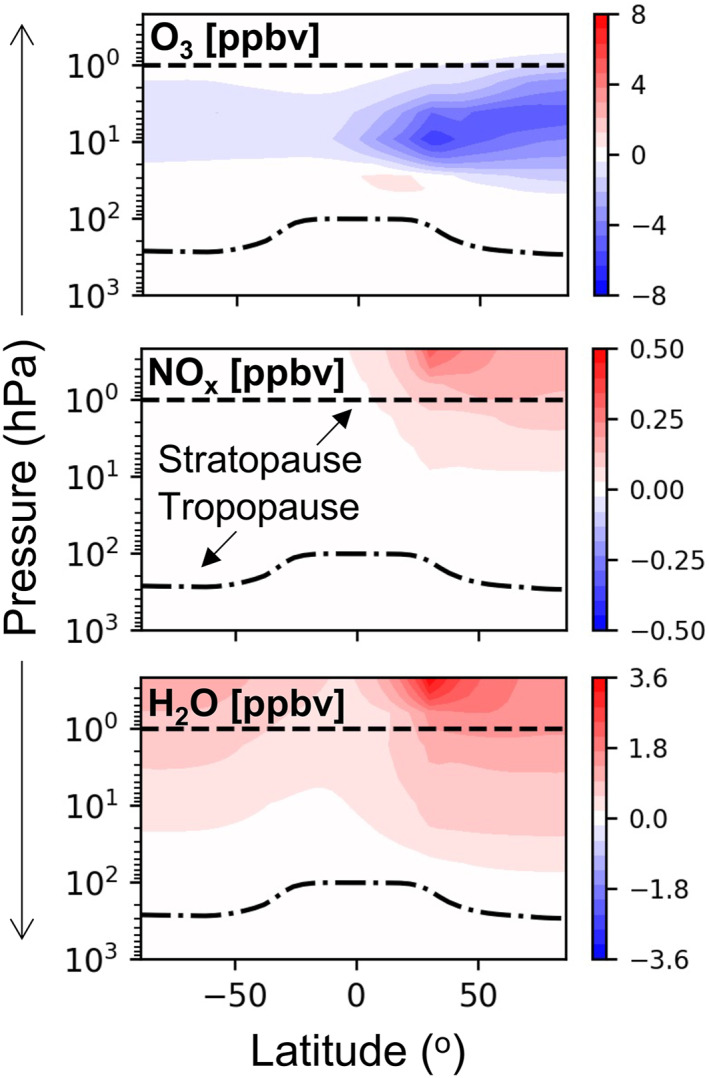
The effect of 3 years of space tourism emissions on atmospheric composition. Panels show the change in annual zonal mean mixing ratios of O_3_, NO_x_, and H_2_O due to rocket emissions.

Figure 7 shows the impacts of projected space tourism emissions on stratospheric chemistry. Spatial and seasonal variability in O_3_, NO_x_ and H_2_O is similar to the contemporary rockets simulation (Figure 3). We simulate 3 years of space tourism, and are able to linearly project the decade long impact from this as the results in Figure 4 show that changes in O_3_, NO_x_ and H_2_O are linear with time once the atmosphere reaches steady state. The magnitude of maximum change in the northern hemisphere upper stratosphere is 3–4 ppbv greater for O_3_ after 3 years of space tourism emissions than a decade of sustained contemporary launch and re‐entry heating emissions. NO_x_ causes almost all the additional O_3_ depletion in the space tourism scenario, as indicated by the sensitivity simulations (Figure 5) and lack of additional chlorine emissions from space tourism rockets. There is no substantial change in the vertical distribution of increases in NO_x_ and H_2_O compared to the 2019 rocket emissions results (Figure 3).

**Figure 8:**
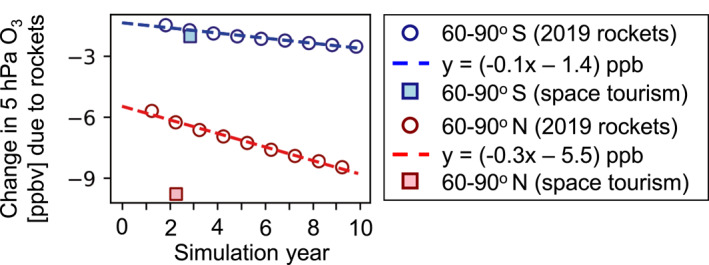
High‐latitude springtime upper stratospheric O_3_ loss due to rocket launch and re‐entry emissions. Open circles show the O_3_ response to rocket emissions at 60–90° and 5 hPa altitude for years 2–10 of the decade‐long 2019 rocket emissions inventory simulation. Dashed lines show the linear least squares fit to these results. Filled squares show the springtime O_3_ loss due to space tourism emissions at the same altitude and latitude range.

Figure 8 contextualizes the upper stratospheric springtime polar O_3_ loss due to space tourism emissions in year 3 by comparison to its decline after a decade of growth in 2019 emissions. We focus on the poles (60–90°) to assess the influence of rocket air pollutant emissions on O_3_ recovery achieved with a global phase‐out of surface sources of O_3_ depleting substances by the Montreal Protocol. O_3_ loss in spring at 60–90°N reaches 5.7 ppbv after the atmosphere equilibrates and continues to decline at a rate of 0.3 ppbv a^−1^ due to sustained 5.6% a^−1^ growth in 2019 emissions. At the end of the decade, O_3_ loss reaches 8.5 ppbv or 0.15%. This is 10% of the O_3_ recovery of ∼81 ppbv dec^−1^ estimated to have been achieved with the Montreal Protocol (Eyring et al., 2010). As the regional temporal change in polar stratospheric O_3_ is linear, we estimate O_3_ depletion after a decade of space tourism emissions using the rate of depletion from the decade‐long contemporary rockets simulation. We find that a decade of space tourism emissions could cause O_3_ depletion at 5 hPa and 60–90°N of ∼13 pbbv. This is 16% of policy‐driven upper stratospheric O_3_ recovery. At 60–90°S, O_3_ depletion of 2.8 ppbv is much less than the recovery due to controls on O_3_ depleting substances (114 ppbv dec^−1^ according to Eyring et al. (2010)).

Figure 6 also shows top‐of‐the‐atmosphere instantaneous radiative forcing due to 3 years of space tourism and 2019 emissions. The space tourism scenario mean global climate forcing is +7.9 mW m^−2^, double the effect of a decade of contemporary emissions and also dominated by BC (+7.7 mW m^−2^). SpaceX space tourism flights account for 52% of all BC emissions. The contribution from Virgin Galactic is 21%. Forcing peaks at +30 mW m^−2^ in the Arctic (Figure S6 in Supporting Information S1). The offset from an increase in PSCs and decline in O_3_ and CH_4_ is near‐negligible (just −0.2 mW m^−2^). Ross et al. (2010) estimated, to first order, a steady state global mean radiative forcing of +43 mW m^−2^ from 1000 annual rockets similar to that used by Virgin Galactic: an air launch and hydrocarbon‐based rocket fuel synonymous with HTPB. This is 35 mW m^−2^ more than our estimate, despite smaller annual emissions of 0.6 versus 1.5 Gg in our scenario. Part of the discrepancy is due to differences in the radius, persistence and optical properties of BC. They estimated a BC loading that is 4.4 times greater than their BC emissions, whereas the BC loading in our space tourism simulation is 1.9 times our BC emissions. Our results for BC lifetime are in the range of results reported by Kravitz et al. (2012) who found a lifetime of 1.4 years for BC with 0.08 μm radius, and 3.8 years for BC with 0.03 μm radius. BC radiative forcing for the space tourism scenario increases the contribution of rocket launch BC emissions to forcing from all BC sources from 3% to 6%. The contribution to total global BC emissions doubles, but is still very small (∼0.02%). BC forcing normalized by emissions is 7,800 mW m^−2^ a^−1^ (Tg BC)^−1^ for contemporary rockets only and 9,900 mW m^−2^ a^−1^ (Tg BC)^−1^ with space tourism launches, exceeding that from all other sources (20.7 mW m^−2^ a^−1^ (Tg BC)^−1^) by a factor of 375 for contemporary rockets and 475 with space tourism. BC forcing is 8 times greater than the absolute BC forcing (0.94 mW m^−2^) from aviation (Lee et al., 2021), even though the cumulative distance traveled by all rockets in the space tourism scenario (∼140,000 km) is 10^5^ times less than that of commercial aircraft in 2019 (61 billion km; Lee et al. (2021)).

## 4 Conclusions

The space sector has evolved markedly since the early space race between the US and the Soviet Union due to establishment of space launch facilities in many countries, technological breakthroughs, commercial space launches, and now even space tourism. Substantial growth is anticipated, necessitating improved understanding of the impact on stratospheric ozone (O_3_) and climate. Here we develop inventories of dominant air pollutant emissions from rocket launches and re‐entry heating of reusable and discarded rocket components and space debris for the modern space sector (2019), for sustained modest (5.6% a^−1^) growth in emissions, and for a speculative space tourism industry. These we incorporated in the 3D atmospheric CTM GEOS‐Chem coupled to a radiative transfer model.

The greatest impact of a decade of emissions on O_3_ occurs in the upper stratosphere in the northern high latitudes. Loss rates in that part of the atmosphere in springtime are 0.15% for 2019 emissions and 0.24% with space tourism emissions, due mostly to NO_x_ from re‐entry heating (51%) and chlorine from solid rockets (49%). A future industry with sustained growth in rocket launches, continued accumulation of space debris, ongoing use of solid rocket fuel, and routine space tourism launches could substantially offset remediation of upper stratospheric O_3_ achieved with the Montreal Protocol.

Warming due to black carbon (BC) is 3.9 mW m^−2^ from a decade of contemporary rockets, dominated by emissions from kerosene‐fueled rockets. This more than doubles (7.9 mW m^−2^) after just 3 years of additional emissions from space tourism launches, due to the use of kerosene and hybrid synthetic rubber fuels. A 7.9 mW m^−2^ warming is 6% of warming due to BC from all other sources, even though the contribution to global BC emissions is 0.02%, as BC directly injected to the upper atmosphere has a greater climate forcing efficiency than other sources. We estimate this to be almost 500 times more than all other BC sources.

Large uncertainties need to be addressed to further enhance our understanding of the true impact of contemporary rocket launch and re‐entry heating emissions on atmospheric composition and climate. These include the size of the nascent space tourism industry and growth in traditional rocket launches and returning space debris, improved estimates of BC emission factors from hybrid synthetic rubber fuels, precise geolocation and mass of space debris re‐entering the Earth's atmosphere, emission factors for other potentially hazardous chemicals formed during rocket launches and re‐entry, improved parameterization of re‐entry heating NO_x_ emissions for returning reusable components, the phase, size distribution and optical properties of alumina (Al_2_O_3_) from solid fuel, and exacerbation of greenhouse gas warming of the troposphere on stratospheric cooling and subsequent depletion of stratospheric O_3_. These uncertainties and the results we obtain support the need to develop international regulation to mitigate environmental harm caused by launch and re‐entry emissions of a fast‐growing industry.

## Conflict of Interest

The authors declare no conflicts of interest relevant to this study.

## Supporting information

Supporting Information S1

## Data Availability

Data used in this study are publicly available from the UCL Data Repository (https://doi.org/10.5522/04/17032349).
