# Supplementary material for: Impact of Rocket Launch and Space Debris Air Pollutant Emissions on Stratospheric Ozone and Global Climate
Source: Earths Future. 2022 Jun 24;10(6):e2021EF002612. doi: 10.1029/2021EF002612 (PMC9287058; doi:10.1029/2021EF002612)
Supplement: Supplementary file 1 — Supporting Information S1 [file EFT2-10-0-s001.docx]

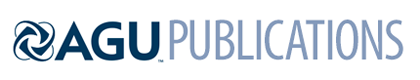


*Earth’s Future*

Supporting Information for

**Impact of rocket launch and space debris air pollutant emissions on stratospheric ozone and global climate**

Robert G. Ryan^1*^, Eloise A. Marais^1^, Chloe J. Balhatchet^2^, Sebastian D. Eastham^3^

*^1^ Department of Geography, University College London, London, UK*

*^2^ Yusuf Hamied Department of Chemistry, University of Cambridge, Cambridge, UK*

*^3^ Laboratory for Aviation and the Environment, Department of Aeronautics and Astronautics, Massachusetts Institute of Technology, Cambridge, MA, USA*

** Corresponding author: robert.ryan@ucl.ac.uk*

**Introduction**

This Supporting Information document contains details of the rockets incorporated in our 2019 emissions inventory and referenced in the main manuscript. These include fuel types, launch locations and the number of launches in Table S1. Figures S1 and S2 show time series of annual rocket launches and re-entries pertinent to the discussion of trends in these in the main text. Figures S3-S5 present additional GEOS-Chem simulation results of the effects of rocket air pollutant emissions on polar stratospheric clouds (Figures S3), the chemical composition of the polar upper stratosphere (Figure S4), and the spatial distribution of the radiative forcing due to contemporary (2019) and speculative space tourism rocket and re-entry ablation emissions (Figure S5). The manuscript includes details of the GEOS-Chem simulations conducted with these emissions to estimate the impact of rocket and re-entry ablation emissions on the atmosphere.

**S1. Overview of rockets launched in 2019**

Table S1 details each of the rockets launched in 2019, including launch countries and propellant types for each launch stage. Rockets with limited data of the mass of propellant used in each stage include the four-stage rockets CZ-11, Hyperbola-1, OS-M1, Jielong-1, Kuaizhou-1A, and Soyuz Volga. We estimate propellant mass for each stage using propellant mass ratios of rockets with comparable size, number of stages and propellant types. These include Vega for CZ-11 and Hyperbola-1, Pegasus-XL for the first three stages and Vega for the final stage of OS-M1 and Jielong-1, the mean of all four-stage solid rockets for the final stage mass of Kuaizhou-1A (values of the first three stages are available), and average first stage masses of all other Soyuz vehicles for Soyuz Volga.

Table S1. Overview of rockets launched in 2019

| Rocket Name | Launch Site | Launches | Propellant (Stages) ^a^ | Reference |
| --- | --- | --- | --- | --- |
| Falcon Series | USA | 13 | Kerosene (1,2,3) ^b^ | (SpaceX, 2020) |
| Delta-4H | USA | 1 | Hydrogen (B,1,2) ^c^ | (United Launch Alliance, 2020b) |
| Delta-4M | USA | 2 | Hydrogen (1,2) ^c^ Solid (B) ^d^ | (United Launch Alliance, 2020b) |
| Antares Series | USA | 2 | Kerosene (1) Solid (2) | (Northrop Grumman, 2020) |
| Atlas V | USA | 2 | Kerosene (1) Hydrogen (2) Solid (B) | (United Launch Alliance, 2020a) |
| Pegasus-XL | USA | 1 | Solid | (Mosier and Rutkowski, 1993) |
| Ariane 5 Series | French Guiana | 4 | Hydrogen (1,2) Solid (B) | (Arianespace, 2020) |
| Vega | French Guiana | 2 | Solid (1,2,3) Hypergolic (4) ^e^ | (Arianespace, 2014) |
| Soyuz Series | French Guiana, Russia, Kazakhstan | 21 | Kerosene (B,2,3) Hypergolic (4) | (Arianespace, 2018), (Starsem, 2001) |
| PSLV Series | India | 4 | Solid (B,1,3) Hypergolic (2,4) | (Indian Space Research Organisation, 2020b) |
| GSLV Mk III | India | 1 | Hypergolic (1) Hydrogen (2) Solid (B) | (Indian Space Research Organisation, 2020a) |
| Electron KS | New Zealand | 6 | Kerosene (1,2) | (Rocket Lab USA, 2020) |
| Epsilon (2) CLPS | Japan | 1 | Solid (1,2,3) Hypergolic (4) | (Japan Aerospace Exploration Agency, 2018) |
| H-2B-304 | Japan | 1 | Hydrogen (1,2) Solid (B) | (Japan Aerospace Exploration Agency, 2020) |
| Proton-M Series | Kazakhstan | 5 | Hypergolic (1,2,3)  Kerosene (4) | (International Launch Services, 2017) |
| Rokot-KM | Russia | 2 | Hypergolic (1,2,3) | (Eurocket, 2011) |
| CZ-2C, CZ-2D | China | 2 | Hypergolic (1,2) | (China Great Wall Industry Corporation, 2020) |
| CZ-3B, CZ-3C | China | 12 | Hypergolic (B,1,2) Hydrogen (3) | (China Great Wall Industry Corporation, 2020) |
| CZ-4B, CZ-4C | China | 7 | Hypergolic (1,2,3) | (Song-An and Jun, 1999) |
| CZ-5 | China | 1 | Kerosene (B) Hydrogen (1,2) | (Kyle, 2020, China Great Wall Industry Corporation, 2020) |
| CZ-6 | China | 1 | Kerosene (1,2,3) | (Kyle, 2020) |
| CZ-11 | China | 3 | Solid (1,2,3,4) | (China Space Report, 2020) |
| Jielong-1 | China | 1 | Solid (1,2,3,4) | (Lia, 2019) |
| Hyperbola-1 ^f^ | China | 1 | Solid (1,2,3) Hypergolic (4) | (I-Space, 2020) |
| Kuaizhou-1A | China | 5 | Solid (1,2,3) Hypergolic (4) | (Kyle, 2020) |
| OS-M1 | China | 1 | Solid (1,2,3,4) | (Kyle, 2020) |
| Simorgh, Safir-1B | Iran | 3 | Hypergolic (1,2) | (Kyle, 2020) |
|  |  |  |  |  |

^a^ Numbers in parentheses indicate fuel type for each rocket stage and for the boosters (B), where used. ^b^ Kerosene fuel uses liquid oxygen as oxidizer. ^c^ Liquid hydrogen fuel uses liquid oxygen as oxidizer. ^d^ Solid rocket propellants use aluminium powder as fuel and ammonium perchlorate as oxidizer. ^e^ Hypergolic propellants use a hydrazine derivative as fuel and dinitrogen tetroxide as the oxidizer. ^f^ Final stage is a liquid propellant that we assume to be hypergolic based on common use of this over kerosene.


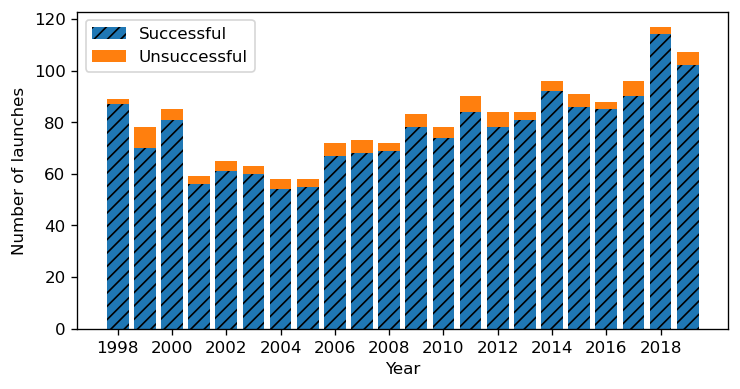


**Figure S1**. Total annual rocket launches from 1998 to 2019. Colours discern successful (blue) and failed (orange) launches. From data compiled by Kyle (2020).


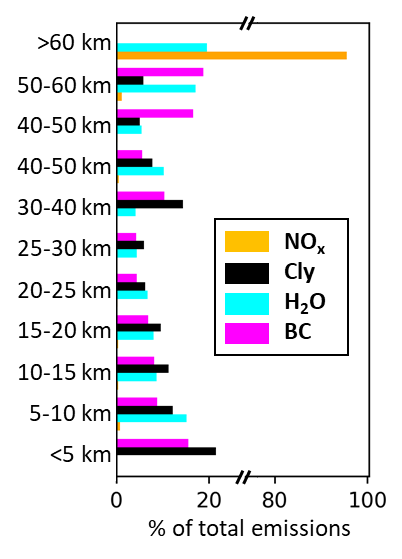


Figure S2. Vertical distribution of rocket pollutant emissions in GEOS-Chem. Bars show the percentage of the total emissions for each pollutant that are emitted in each altitude range. Note that the bin sizes on the y-axis vary, and that Al_2_O_3_ emissions are distributed in the same way as Cl_y_ (Cl + HCl).


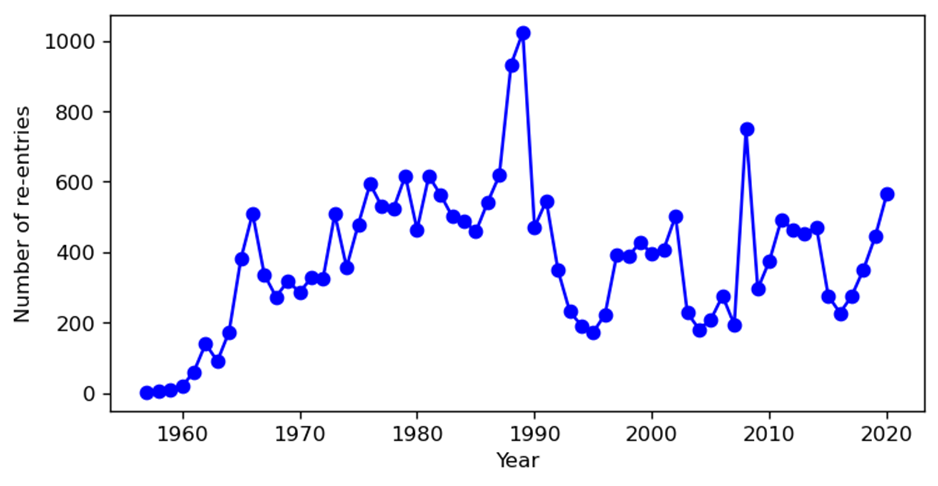


**Figure S3**. Number of objects re-entering the atmosphere from space since the start of the space race. From data compiled by the European Space Agency (<https://discosweb.esoc.esa.int/>; last accessed 4 May 2022).


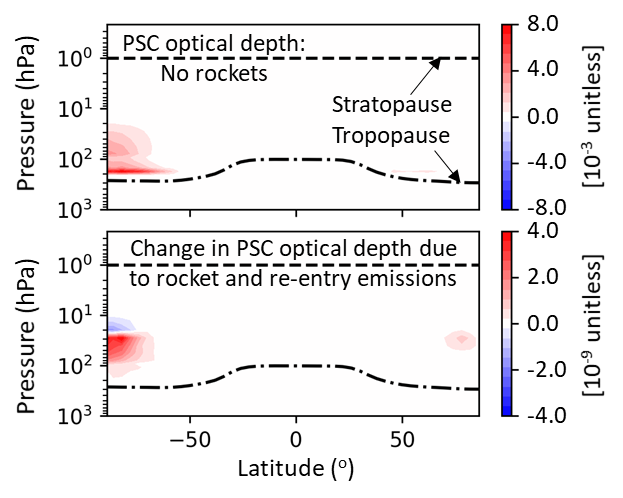


Figure S4. Influence of a decade of rocket and re-entry emissions on polar stratospheric clouds (PSCs). Panels are GEOS-Chem absolute optical depth due to PSCs in the absence of rocket launch and ablation emissions (top) and the difference in optical depth for a simulation with and without these emissions (bottom).


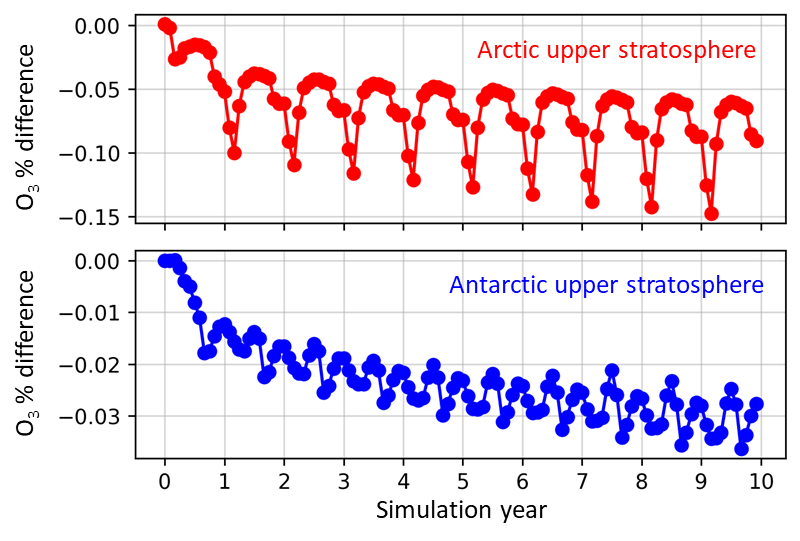


Figure S5. Response of polar upper stratospheric composition to rocket air pollutant emissions over 10 years. Points show the percentage difference in mean mixing ratio between GEOS-Chem simulations with and without 2019 rocket and re-entry emissions (with minus without) for grids centered on 5 hPa (3-6 hPa) for ozone (O_3_), methane (CH_4_), nitrogen oxides (NO_x_) and total inorganic chlorine (Cl_y_) at polar latitudes (60-90^o^N in red, 60-90^o^S in blue).

**
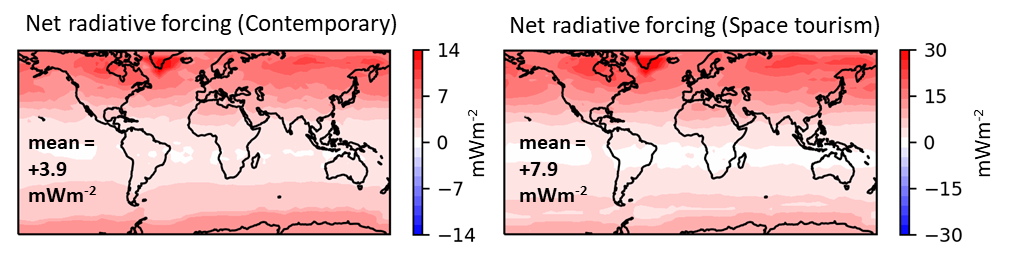
**

Figure S6. The effect of rocket launch and re-entry emissions on global radiative forcing. Panels show the spatial distribution of radiative forcing changes after a decade of growth in contemporary (2019) emissions (left) and from the space tourism scenario (right).
